# Regulation of PGC-1α mediated by acetylation and phosphorylation in MPP+ induced cell model of Parkinson’s disease

**DOI:** 10.18632/aging.103219

**Published:** 2020-05-26

**Authors:** Fei Fan, Songlin Li, Zhipeng Wen, Qiaoyue Ye, Xiaochun Chen, Qinyong Ye

**Affiliations:** 1 Department of Neurology, Fujian Institute of Geriatrics, Fujian Medical University Union Hospital, Fuzhou, Fujian, China; 2 Fujian Health College, Fuzhou, Fujian, China; 3 Affiliated Sichuan Provincial Rehabilitation Hospital of Chengdu University of TCM, Sichuan Bayi Rehabilitation Center, Chengdu, Sichuan, China; 4 Affiliated Hospital of Putian University, Putian, Fujian, China; 5 Fuzhou No. 8 High School, Fuzhou, Fujian, China; 6 Institute or Neuroscience, Fujian Key Laboratory of Molecular Neurology, Fujian Medical University, Fuzhou, Fujian, China

**Keywords:** Parkinson’s disease, GCN5, p38MAPK, AMPK, PGC-1α

## Abstract

Background: Parkinson’s disease (PD) is one of the most common neurodegenerative diseases with complex etiology in sporadic cases. Accumulating evidence suggests that oxidative stress and defects in mitochondrial dynamics are associated with the pathogenesis of PD. The oxidative stress and mitochondrial dynamics are regulated strictly by peroxisome proliferator-activated receptor γ coactivator-1α (PGC-1α). We investigated whether acetylation and phosphorylation of PGC-1α contribute to protecting neuronal cell against oxidative stress.

Results: We found that acetylation and phosphorylation mediated the nuclear translocation of PGC-1α protects against oxidative damage. In contrast to the increased nuclear PGC-1α, the cytosolic PGC-1α was decreased upon inhibition of GCN5 acetyltransferase. Similarly to the inhibition of GCN5 acetyltransferase, the increased nuclear PGC-1α and the decreased cytosolic PGC-1α were observed upon p38MAPK and AMPK activation. Briefly, the significantly increased nuclear PGC-1α is regulated either by inhibiting the acetylation of PGC-1α or by the phosphorylating PGC-1α, which results in a reduction in ROS.

Conclusion: PGC-1α protects neuronal cells against MPP^+^-induced toxicity partially through the acetylation of PGC-1α mediated by GCN5, and mostly through the phosphorylation PGC-1α mediated by p38MAPK or AMPK. Therapeutic reagents activating PGC-1α may be valuable for preventing mitochondrial dysfunction in PD by against oxidative damage.

Methods: With established the 1-methyl-4-phenylpyridinium (MPP^+^)-induced cell model of PD, the effects of MPP^+^ and experimental reagents on the cell viability was investigated. The expression of PGC-1α, general control of nucleotide synthesis 5 (GCN5), p38 mitogen-activated protein kinase (p38MAPK) and adenosine monophosphate activated protein kinase (AMPK) were detected by Western blotting and quantitative real-time PCR. The level of reactive oxygen species (ROS) was measured by flow cytometry. All statistical analyses were carried out using one-way ANOVA.

## INTRODUCTION

Parkinson’s disease (PD) is one of the most common neurodegenerative disorders with the loss of dopaminergic neurons in the substantia nigra pars compacta causing a plethora of motor symptoms. Both environmental and genetic factors are involved in the pathogenesis of PD. Massively parallel analysis of messenger RNA transcripts has demonstrated that there is a global change in genes related to PD, of which approximately 10 genes in control of cellular bioenergetics associated with PD are modulated by the peroxisome proliferator-activated receptor γ coactivator-1α (PGC-1α) [1]. As a co-transcriptional regulator, PGC-1α regulates the expression of genes involved in mitochondrial respiratory, the mitochondrial dynamics and biogenesis as well as the oxidative metabolism [2]. Furthermore, increasing lines of evidence suggest that the PGC-1α abrogation is correlated with PD [3–5].

PGC-1α protects dopaminergic neuron from loss induced by mutated α-synuclein or the pesticide rotenone [1] and prevents cell death and axonopathy by regulation of mitochondrial biogenesis, reactive oxygen species (ROS) and detoxification [6, 7]. In addition, the transgenic mice overexpressing PGC-1α in dopaminergic neurons are resistant against cellular degeneration and dopamine loss induced by the neurotoxin 1-methyl-4-phenyl-1,2,3,6-tetrahydropyridine (MPTP), a prodrug to the neurotoxin 1-methyl-4-phenylpyridinium (MPP^+^) causing permanent symptoms of PD [8]. Deletion of PGC-1α gene leads to the degeneration of dopaminergic neurons in the substantia nigra and a concomitant decrease in striatal dopamine [9]. Taken together, PGC-1α plays a key role in protecting cells by the regulation of mitochondrial biogenesis and oxidative stress. However, the mechanism by which how PGC-1α plays its role in PD has not yet been elucidated.

The activity of PGC-1α is affected by multiple post-transcriptional modifications, such as acetylation, phosphorylation and methylation [10–13]. The transcriptional activity of PGC-1α is inhibited upon its acetylation by an acetyltransferase, general control of nucleotide synthesis 5 (GCN5) [14], whilst the reactivation of PGC-1α by deacetylation via suppression of GCN5 protects neurons against huntingtin-induced degeneration through increasing mitochondrial density [15]. Moreover, the transcriptional activity of PGC-1α is able to be enhanced upon its phosphorylation by p38 Mitogen-activated protein kinase (p38MAPK) [16–18] or extracellular signal-regulated kinase1/2 (ERK1/2) [19].

However, the mechanism by which these multiple post-transcriptional modifications regulate PGC-1α function, especially in the dopaminergic neurons, remains largely unknown. In our previous reports, overexpressing PGC-1α or silencing PGC-1α is involved in the mitochondrial protection in MPP^+^-induced cell model of PD [20, 21], and expressing PGC-1α in dopaminergic neurons reverses the effects of MPTP-induced mitochondrial dysfunction in C57BL mice [22]. Thus, the aim of this current study is to further dedicate whether acetylation or phosphorylation of PGC-1α in a cell model of PD can maintain mitochondrial homeostasis to protect neuronal cell under stresses.

## RESULTS

### Establishment of a cell model of PD

**Figure 1:**
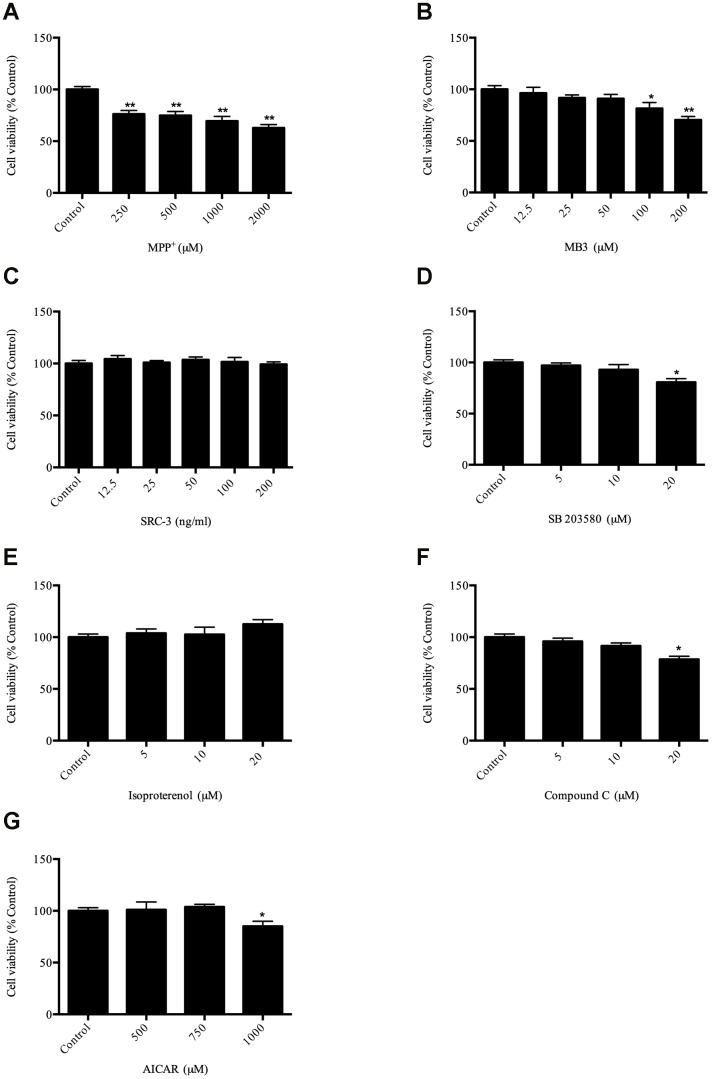
Evaluation of compounds on cell viability (**A**) cell viability after MPP^+^ treatment; (**B**) cell viability after MB-3 treatment; (**C**) cell viability after SRC-3 treatment; (**D**) cell viability after SB203580 treatment; (**E**) cell viability after isoproterenol treatment; (**F**) cell viability after Compound C treatment; (**G**) cell viability after AICAR treatment. * *P* < 0.05, ** *P* < 0.01.

To evaluate the viability of SH-SY5Y cells after treatment with MPP^+^, SH-SY5Y cells were treated with MPP^+^ at different concentrations (250–2000 μM) for 24 h. The survival result clearly showed that MPP^+^ inhibited cell survival in a dose-dependent manner (Figure 1A). According to our result (Figure 1A) and the previous observation [23], the cells exposed to 1000 μM MPP^+^ as an optimal concentration were selected to establish a cell model of PD for subsequent experiments [20]. Next, to determine optimal concentrations of compounds to be used for this study, the cell viability was measured by MTT assay. Based on the MTT assay, the appropriate concentration of reagents were used for subsequent experiments: MB-3 (50 μM), SRC-3 (100 ng/mL), SB203580 (10 μM), isoproterenol (10 μM), Compound C (10 μM), AICAR (500 μM) (Figure 1B–1G).

### Cytosolic rather than nuclear PGC-1α distribution was regulated by GCN5

**Figure 2:**
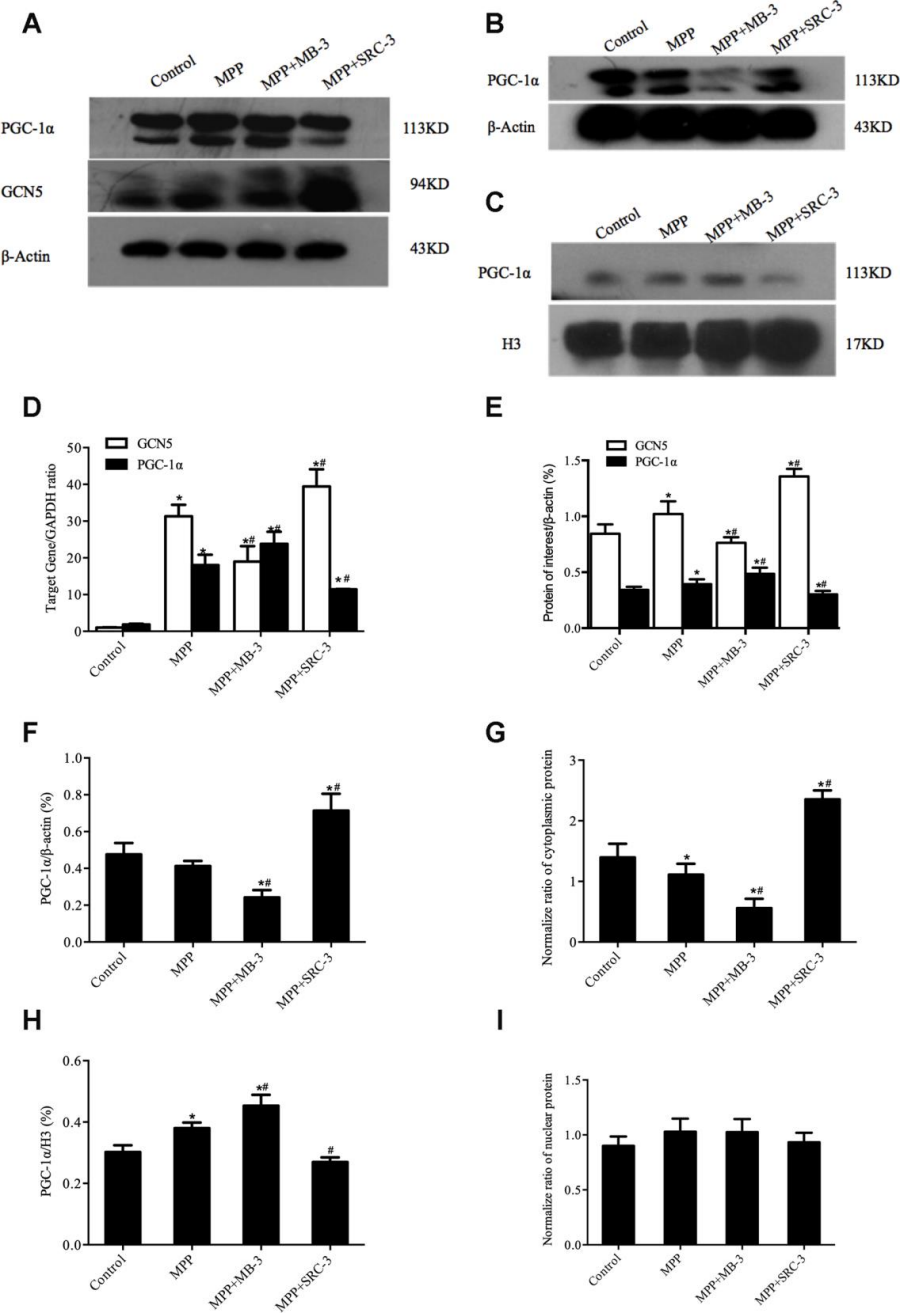
**The cytosolic rather than the nuclear distribution of PGC-1α regulated by GCN5 in an MPP^+^-treated cell model.** (**A**) The protein levels of GCN5 and PGC-1α; (**B**, **C**) The cytosolic levels of PGC-1α (**B**) and the nuclear levels of PGC-1α (**C**); (**D**) The relative transcriptional levels of GCN5 and PGC-1α normalized to GAPDH; (**E**) Semi-quantification of total GCN5 and PGC-1α proteins relative to β-actin; (**F**, **H**) Semi-quantification of the cytosolic (**F**) and the nuclear (**H**) PGC-1α proteins relative to β-actin; (**G**, **I**) The normalized cytosolic (**G**) and nuclear (**I**) proteins relative to the total protein; n=6, per group. * *P* <0.05, *vs.* Control; # *P* <0.05, *vs.* MPP^+^.

To determine whether acetylation of PGC-1α was mediated by GCN5 in the MPP^+^-mediated cell model, we first tested whether inhibition of GCN5 by MB-3 or activation of GCN5 by SRC-3 would affect the levels of mRNA and protein of GCN5 and PGC-1α. After cocultured with MB-3, a GCN5 inhibitor or SRC-3, a GCN5 activator [24, 25] for 48 h, the cells were treated with MPP^+^ (1000 μM) for another 24 h. As shown in Figure 2, upon MPP^+^ treatment, the mRNA levels of GCN5 and PGC-1α were significantly elevated compared with control. Upon MB-3 treatment, the mRNA level of GCN5 was decreased by 39.31% and the mRNA level of PGC-1α was increased by 32.16%, compared to MPP^+^ control, while upon SRC-3 treatment, the mRNA level of GCN5 was increased by 26.02% and the mRNA level of PGC-1α was decreased by 36.50%, compared to MPP^+^ control (Figure 2D). In agreement with the changes of mRNA levels, the protein levels of both GCN5 and PGC-1α were upregulated by 19.59% and by 15.09%, respectively, after only MPP^+^ treatment compared with control. Consistent with the changes of mRNA levels, upon MB-3 treatment, the protein level of GCN5 was decreased by 27.17% and the protein level of PGC-1α was increased by 23.35%, compared to MPP^+^ control, while upon SRC-3 treatment, the protein level of GCN5 was increased by 65.51% and the protein level of PGC-1α was decreased by 23.22%, compared to MPP^+^ control (Figure 2A, 2E). These data demonstrated that the expression of PGC-1α was correlated with GCN5 activity.

Next, we determined whether the distribution of PGC-1α is associated with GCN5 activity. As shown in Figure 2B, 2C, 2F, 2H, the nuclear PGC-1α was significantly increased in response to MPP^+^ treatment compared with control (*P* <0.05). In addition, after MPP^+^ plus MB-3 treatment, the nuclear PGC-1α was increased by 18.01% compared with MPP^+^ (*P* <0.05), while the cytosolic PGC-1α was decreased by 42.04% (*P* <0.05). In contrast, after MPP^+^ plus SRC-3 treatment, the nuclear PGC-1α was decreased by 28.94% compared with MPP^+^ (*P* <0.05), while the cytosolic protein level of PGC-1α was increased by 72.52%. To precisely evaluate the nuclear and the cytosolic distribution of PGC-1α, the nuclear and the cytosolic PGC-1α were normalized to the total protein. The normalized data showed that the cytosolic PGC-1α but not the nuclear PGC-1α was affected by GCN5 activity (Figure 2G, 2I).

### The GCN5-mediated nuclear translocation of PGC-1α reduced ROS levels in MPP^+^ induced cell model of PD

**Figure 3:**
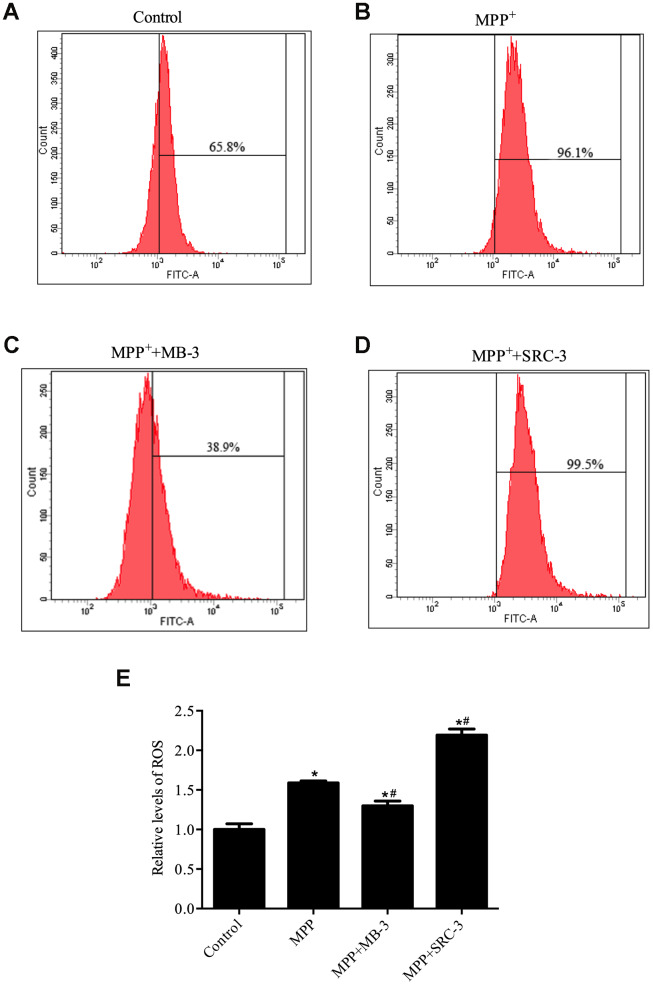
**ROS production was regulated by GCN5 in MPP^+^-treated cell model.** (**A**) Relative levels of ROS in control group; (**B**) Relative levels of ROS in cells treated with MPP^+^ (1000 μM), (**C**) Relative levels of ROS in cells treated with MPP^+^ (1000 μM) and MB-3 (50 μM); (**D**) Relative levels of ROS in cells treated with MPP^+^ (1000 μM) and SRC-3 (100 ng/mL). (**E**) Bar graph of relative levels of ROS; n=6, per group. *P < 0.05 *vs.* control group; # *P* < 0.05 *vs.* MPP^+^ group.

PGC-1α plays an important role in reactive oxygen species (ROS) generation [26]. Therefore, we sought to determine whether manipulation of GCN5 activity with inhibitor MB-3 and activator SRC-3 would affect ROS production in MPP^+^-mediated neuronal cell toxicity model. First, we tested the direct effect of MPP^+^ on ROS production in SH-SY5Y cells. There was an increase of 30.3% in ROS-positive cells when treated by MPP^+^ compared with control (Figure 3). Given that PGC-1α protein is translocated into the nucleus in an early response to oxidative stress, which could attenuate the ROS formation [27] and inhibition of GCN5 by MB-3 causes translocation of PGC-1α from the cytoplasm into the nucleus, we therefore speculated that the number of ROS-positive cells would decrease upon MPP^+^ (1000 μM) pretreatment following MB-3 (50 μM) treatment. Indeed, a significant decrease in ROS-positive cells (57.2%) was observed in a combined treatment with MPP^+^ and MB-3 compared with MPP^+^ treatment only (Figure 3). In contrast, a significant increase in ROS-positive cells (4%) was observed upon MPP^+^ (1000 μM) pretreatment plus SRC-3 compared to MPP^+^ treatment only. Taken together, our data indicated that GCN5 activity directly affects ROS levels in SH-SY5Y cells and the ROS production is possibly regulated by the nuclear translocation of PGC-1α.

### Phosphorylation of PGC-1α was mediated by p38MAPK and AMPK

**Figure 4:**
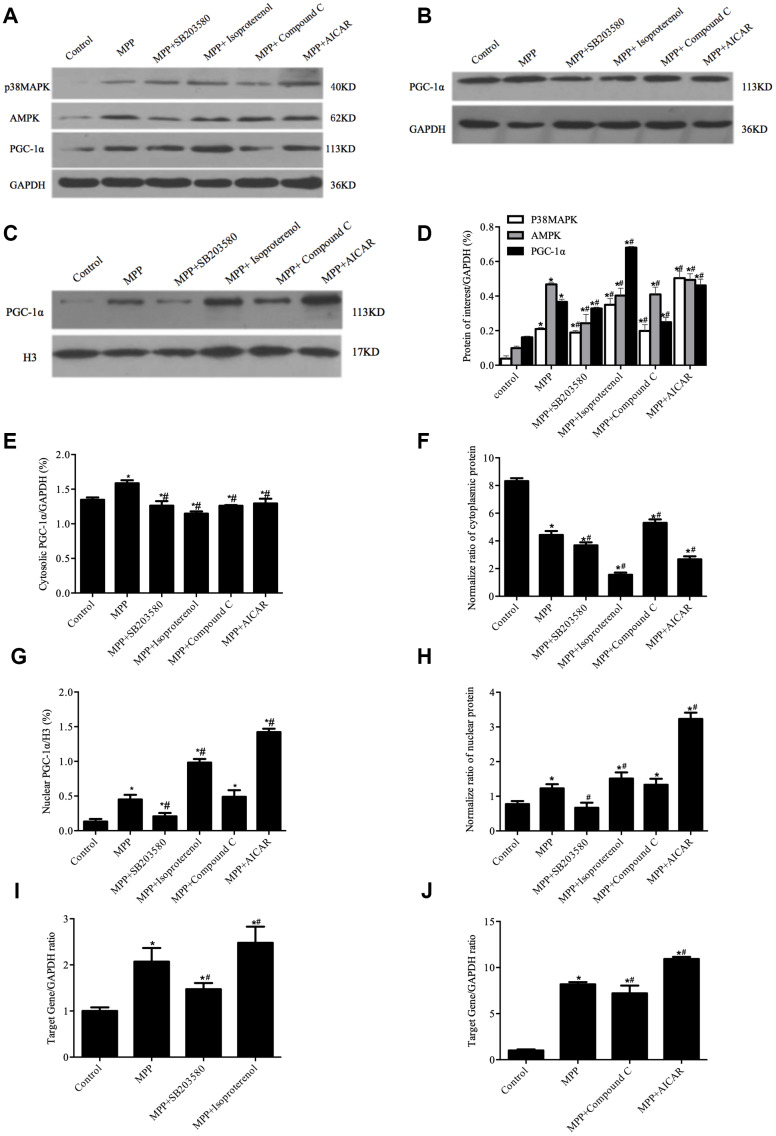
**Redistribution of PGC-1^α^ was regulated by p38MAPK and AMPK in MPP^+^-treated cell model.** (**A**) Protein levels of p38MAPK, AMPK, and PGC-1^α^. (**B**, **C**) Cytosolic (**B**) and nuclear (**C**) protein levels of PGC-1^α^. (**D**) Semi-quantification of total protein levels of p38MAPK, AMPK, and PGC-1^α^ relative to GAPDH; (**E**, **G**) Semi-quantification of cytosolic (**E**) and nuclear (**G**) protein levels of PGC-1^α^ relative to GAPDH or H3; (**F**, **H**) Normalized cytosolic (**F**) and nuclear (**H**) proteins to the total proteins. (**I**, **J**) Transcriptional levels of p38MAPK (**I**) and AMPK(**J**) relative to GAPDH; n=6, per group. *P < 0.05, *vs.* Control group; # *P* < 0.05, *vs.*MPP^+^ group.

Previous studies have demonstrated that the phosphorylation of PGC-1α by p38MAPK leads to the nuclear translocation of PGC-1α [18]. To determine whether p38MAPK or AMPK activity would affect the nuclear translocation PGC-1α in MPP^+^-mediated SH-SY5Y cell toxicity model, the cells were pretreated with p38MAPK inhibitor SB203580 (10 μM), or p38MAPK activator isoproterenol (10 μM), or AMPK inhibitor Compound C (10 μM), or AMPK activator AICAR (500 μM), followed by MPP^+^ treatment. First, the mRNA and protein levels of p38MAPK and AMPK were checked by real-time PCR and Western blotting analyses. In contrast to an increase in the mRNA and protein levels of both p38MAPK and AMPK upon activation of p38MAPK and AMPK, the inactivation of p38MAPK and AMPK led to a decrease in mRNA and protein levels of both p38MAPK and AMPK (Figure 4).

Next, the nuclear and the cytosolic PGC-1α were measured by Western blotting. As shown in Figure 4, compared with MPP^+^ treatment alone, pretreatment with p38MAPK activator isoproterenol or AMPK activator AICAR resulted in an increase of 85.59% or 25.94% in the total level of PGC-1α protein, respectively (Figure 4A, 4D, *P*<0.05) as well as an increase of 78.9% or 215.9% in the nuclear PGC-1α, respectively (Figure 4C, 4G, *P*<0.05) in contrast to a decrease of 27.66% or 22.6% in the cytosolic PGC-1α, respectively (Figure 4B, 4E, *P* < 0.05). In contrast, the pretreatment with p38MAPK inhibitor SB203580 led to a decrease in the total, the cytosolic and the nuclear levels of PGC-1α, whereas the pretreatment with AMPK inhibitor Compound C only caused a decrease in the total and the cytosolic levels of PGC-1α but not the nuclear levels. Of note, the normalized data demonstrated that the nuclear and the cytosolic levels of PGC-1α changed in opposite directions in response to the treatment with different compounds (Figure 4F, 4H, *P*<0.05). Together, these data indicated that phosphorylation of PGC-1α by p38MAPK or AMPK directed the redistribution of PGC-1α.

## DISCUSSION

Given that mitochondria is vital in the cellular energy metabolism, it is not surprising that mitochondrial dysfunction contributes to the pathogenesis of PD. PGC-1α has emerged as a major player in regulation of mitochondrial biogenesis, leading to increased mitochondrial mass, enhanced mitochondrial respiratory function and upregulated antioxidant defense status, which shed light on the protective roles of PGC-1α in pathogenesis of PD. In support of that notion, it has been evident that the stabilized full length PGC-1α by Necdin (a melanoma antigen family protein) promotes mitochondrial biogenesis to exert neuroprotection against mitochondrial insults in PD model [28–31]. In neurons, PGC-1α is normally located in both the nucleus and the cytoplasm. Neuronal depolarization induces a significant increase in PGC-1α expression in the cytoplasm facilitating the translocation of PGC-1α into the nucleus for downstream genes activation [32]. Therefore, the subcellular distribution of PGC-1α may also play an important role in mitochondrial function and in pathogenesis of PD.

Indeed, posttranslational modification of proteins plays an essential role in protein translocation. The growing evidence suggests that the acetylation and the phosphorylation of PGC-1α can destine its subcellular distribution to undergo its mitochondrial function. A recent study has confirmed that the acetylation of PGC-1α by acetyltransferase GCN5 can regulates hepatic glucose metabolism [33], by which GCN5 is recruited to its co-activator SRC-3 [34] to facilitate the acetylation of PGC-1α [35]. In this study, we further confirmed that SRC-3 enhances the expression of GCN5 in the dopaminergic neurons, which can be inhibited by MB-3 in turn to affect the acetylation of GCN5-regulated proteins [36]. On the other hand, our study also demonstrated that MB-3 can significantly inhibit the expression of GCN5 by blocking GCN5 activity, indicating that MB-3 can be potentially used as a specific inhibitor of GCN5 to reduce both mRNA and protein levels of GNC5. Since the translocation of PGC-1α to nucleus can be regulated by GNC5-mediated acetylation, upon MPP^+^treatment, we postulated that the increased PGC-1α protein plays a protective role against oxidative stress through the nuclear redistribution of PGC-1α to activate survival genes. Here, we demonstrated an increase in the total and the nuclear PGC-1α, in contrast to the cytosolic one in cells treated with MPP^+^ and MB-3 compared to MPP^+^ treatment alone. However, there is a decrease of 42.2% in the cytosolic PGC-1α, albeit no change in the nuclear PGC-1α after normalization of the nuclear and the cytosolic PGC-1α to the total protein. Taken together, our data indicate that the increase in the nuclear PGC-1α is not only due to de novo synthesis, but partially also due to the redistribution of PGC-1α from the cytoplasm after inhibition of GCN5. Accordingly, the redistribution of PGC-1α to nucleus underlying the activation on survival genes plays a critical role in protecting cells against death. Notwithstanding that the acetylation of PGC-1α by GCN5 has been stated to play an important role in maintaining mitochondrial homeostasis, its precise mechanisms need to be further explored completely in the future because of no antibody specific for acetylated PGC-1α.

Taking into account the fact that both PGC-1α and GCN5 play an important role in regulation of ROS [26, 37, 38] and GCN5 can regulate the redistribution of PGC-1α, it is likely that the GCN5/PGC-1α pathway plays an important role in maintaining ROS homeostasis. Consistently, our data support that GCN5-mediated PGC-1α acetylation affects ROS level in cells treated with MPP^+^, by which ROS stress triggers PGC-1α expression to protect cells from oxidative stress and cell death. However, MPP^+^-induced cellular stress also activates GCN5 expression, which plays a negative role in PGC-1α activation. How does cell determine its fate in the MPP^+^ induced cell model of PD? It possibly depends on whether activated PGC-1α could overcome the GCN5 effect. Actually, our previous study has unveiled that the increased redistribution of PGC-1α into nucleus significantly ameliorates the anti-oxidative stress ability of PGC-1α by inhibition of GCN5 acetylation activity. Therefore, GCN5/ PGC-1α pathway plays a regulatory role in protecting dopaminergic neurons from oxidative stress. The recent study showed that over-expression of SIRT1, which leads to an increase in its deacetylase activity, protects SH-SY5Y cells through [39] upregulation of PGC-1α transcriptional activity [40]. However, it still remains to be determined in the future whether the deacetylation of PGC-1α through SIRT1 is required for the redistribution of PGC-1α.

P38MAPK dysregulation is associated with multiple pathophysiological processes, such as inflammation, cellular stress, and cellular apoptosis [41, 42]. Our data revealed that manipulating p38MAPK activity significantly affects PGC-1α subcellular distribution. Meanwhile, AMPK widely regulates various cellular processes and have broad neuroprotective effects [43]. Activation of AMPK by its activator AICAR leads to an increase in PGC-1α protein levels [44]. On the contrary, inactivation of AMPK by compound C results in a decrease in PGC-1α protein levels [45]. Somewhat differently, but consistent with previous studies, our data showed a significant decrease in the cytosolic PGC-1α in contrast to an increase in the total PGC-1α and in the nuclear PGC-1α upon MPP^+^ pretreatment following Isoproterenol or AICAR treatment compared to MPP^+^ treatment alone after normalized PGC-1α to the total protein, indicating that the nuclear PGC-1α is mostly translocated from cytoplasm after activation of AMPK or p38MAPK. Recently, Ng et al. reported that AMPK-mediated neuroprotection appears to require PGC-1α in a Parkin null drosophila model [46], in which AMPK directly phosphorylates PGC-1α at Threonine-177 and Serine-538 [11]. On the other hand, the phosphorylation of PGC-1α at Threonine 262, Serine 265, and Threonine 298 by p38MAPK activates PGC-1α [17], in combination with our data on p38MAPK-mediated redistribution of PGC-1α, suggesting that the phosphorylation of PGC-1α by p38MAPK not only stabilizes but also redistributes PGC-1α to activate neuroprotective genes. Furthermore, p38MAPK also upregulates PGC-1α gene expression via phosphorylation of ATF-2 at Threonine 71, subsequently, ATF-2 binds to the cAMP-response element-binding protein site on the PGC-1α promoter to induce PGC-1α transcription [18, 47]. In addition, crosstalk between AMPK and p38MAPK plays an important role in mitochondrial functions regulated by PGC-1α [48]. Accordingly, it is interesting to study the roles of different phosphorylation sites of PGC-1α in PD in the future.

## CONCLUSIONS

Our findings elucidated that GCN5 and AMPK/p38MAPK regulate the acetylation and the phosphorylation of PGC-1α. It is likely that the phosphorylation of PGC-1α may play a more important role than acetylation of PGC-1α, by which the transcriptional activity of PGC-1α is tightly controlled in MPP^+^-induced cell toxicity model. Therefore, other pathways associated with deacetylation should be studied in the future to clarify whether deacetylation of PGC-1α is critical for neuronal protection. Nevertheless, the regulatory pathways of PGC-1α, identified in this study, suggest that therapeutic reagents activating PGC-1α may be valuable for repurposing to treat PD.

## MATERIALS AND METHODS

### Reagents

Cell culture reagents (DMEM/F12, fetal bovine serum, penicillin, and streptomycin) were purchased from HyClone (Logan, UT, USA). MPP^+^(No. D048), MTT (No. M2128), SB203580 (No. S8307), isoproterenol (No. I5627), Compound C (No. P5499), and AICAR (No. A9978) were purchased from Sigma-Aldrich (St. Louis, MO, USA). GCN5 inhibitor, MB-3 (No. ab141255) and GCN5 activator, SRC-3 (No. ab4915) were purchased from Abcam Company (Abcam, Cambridge, MA, USA). These reagents were dissolved in dimethyl sulfoxide, and finally diluted with DMEM/F12 to defined concentrations. Primary antibody for PGC-1α (No. ST1202-1SETCN) was purchased from Millipore (EMD Millipore, Billerica, MA, USA). Primary antibodies for H3 (No. ab8896), GCN5 (No. ab181068), p38MAPK (No. ab197348), and AMPK (No. ab3759) were purchased from Abcam (Abcam, Cambridge, MA, USA). Antibodies for Actin and GAPDH, goat anti-rabbit IgG, and goat anti-mouse IgG were purchased from Beyotime Institute of Biotechnology, Jiangsu of China. All other reagents were purchased from Beyotime Company of Biotechnology, Jiangsu of China.

### Cell culture

The human SH-SY5Y neuronal cell line was obtained from the Chinese Academy of Sciences Committee Type Culture Collection cell bank and cultured in Dulbecco’s modified Eagle’s (DMEM)/F12 medium supplemented with 10% fetal bovine serum, 100 mg/mL streptomycin, and 100 units/mL penicillin, in a humidified incubator at 37°C (Forma Scientific, OH, USA; model No. 3130), containing 5% CO_2_. Cells at 60%-70% confluency were pretreated with either inhibitors or activators in DMEM/F12 medium for 48 h, and then treated with MPP^+^ for 24 h. Control cells were only treated with 1%DMSO (vehicle).

### MTT assay

SH-SY5Y cells were seeded on 96-well plates at a density of 1×10^4^ cells/well, cultured for 24 h, and treated with different concentrations of MPP^+^, Butyrolactone 3 (MB-3), SRC-3, SB203580, isoproterenol, Compound C, or AICAR. A total of 20 μL of MTT (0.5 mg/mL) was added to the media (200 μL) in each well. The plates were incubated for 4 h at 37 °C, the MTT-media solution (220 μL) was removed, and 150 μL of dimethyl sulfoxide was added to each well. Reduced MTT was measured on an ELISA reader (Bio-Rad, USA) at a wavelength of 570 nm. Values for each treatment group are expressed as a percentage of the control.

### Measurements of ROS levels

Cells were seeded on 25 cm^2^ culture flasks for 24 h and treated with different reagents. Intracellular ROS levels were determined with DCFH-DA (Beyotime Institute of Biotechnology, China). For a negative control, cells were incubated with PBS alone. For a positive control, cells were incubated with PBS, 1 mL DCFH-DA (10 μM), and 2 μL ROS-UP. After a 20-min incubation, cells were trypsinized, washed, and resuspended in PBS. The levels of fluorescence were immediately detected using fluorescence-activated cell sorter Aria (Becton Dickinson, USA).

### Quantitative real-time PCR analysis

Total RNA from SH-SY5Y cells was isolated according to the manufacturer’s protocol using Trizol reagent (Invitrogen, Carlsbad, CA, USA). Total RNA purity and integrity was confirmed using the ND-1000 NanoDrop (NanoDrop Technologies) and 2100 Bioanalyzer (Agilent, Santa Clara, CA, USA). RNA (1 μg) was reverse-transcribed to complementary DNA (cDNA) in a total volume of 20 μL using the RevertAidTM First Strand cDNA Synthesis Kit (Fermentas, St. Leon-Rot, Germany). The cDNA (2 μL) was amplified with a sequence detection system (ABI PRISM 7500) in a total volume of 20 μL containing 10 μL of the FastStart Universal SYBR Green Master Mix (ROX) (Roche, Germany). Forward and reverse primers were designed to eliminate the possibility of amplifying genomic DNA and the primer sequences are:

PGC-1α (Forward, 5′-ACACAGTCGCAGTCACAACAC-3′ and Reverse, 5′-GCAGTTCCAGAGAGTTCCACA-3′); GCN5 (Forward, 5′-TGGAGAGCGTTCCTGGCATTC-3′ and Reverse, 5′-GGAAGCGGATGACCTCGTAGTAGT-3′); AMPK (Forward, 5′-ATTCGGAGCCTTGATGTG-3′; Reverse, 5′-CCAGCCTTCCATTCTTACAG-3′); p38MAPK (Forward, 5′-ACCTACAGAGAACTGCGGTTAC-3′and Reverse, 5′-TGAGATGGGTCACCAGATACAC-3′); GAPDH (Forward, 5′-AGAAGGCTGGGGCTCATTTG-3′ and Reverse, 5′-AGGGGCCATCCACAGTCTTC-3′). Quantitative real-time PCR was performed using the ABI PRISM7500 HT sequence detection system (Applied Biosystems, Foster City, CA) based on the 59-nuclease assay for the various genes indicated and the housekeeping gene GAPDH. Relative expression was calculated using the ΔΔCt method, after passing the validation experiment. Results are expressed as an average of triplicate samples of at least three independent experiments for control and treated cells.

### Western blotting analysis

After treated with different reagents, cells were washed and, harvested in Radio Immuno Precipitation Assay (RIPA) buffer containing Phenylmethanesulfonyl fluoride (PMSF) (Beyotime Institute of Biotechnology, China), incubated for 10 min on ice, centrifuged at 12,000×g for 10 min at 4°C, and supernatant was collected. Equal amounts of protein (45 μg) were separated by 8% sodium dodecyl sulfate-polyacrylamide gel electrophoresis (SDS -PAGE), then transferred electrophoretically onto polyvinylidene fluoride (PVDF) membrane (Millipore, Ireland). The blots were blocked by incubation in 5% (w/v) non-fat dry milk in PBS with 0.1% Tween 20 (PBS-T) for 3 h. After incubation with various primary antibodies, anti-PGC-1α (1:1000), or anti-GCN5 (1:2000), or anti-p38MAPK (1:1000), or anti-AMPK (1:1000), or β-actin (1:2000), or anti-H3 (1:1000), or GAPDH (1:1500) in PBS-T at 4°C overnight, the PVDF membranes were washed three times in PBS-T for 10 min. Subsequently, the membranes were incubated for 1.5 hour in PBS-T containing secondary antibody conjugated to horseradish peroxidase (anti-mouse IgG (1:2000) or anti-rabbit IgG (1:2000)). The immunoreactive bands were visualized and quantified using the Luminata ForteWestern HRP substrate (Millipore, USA). Protein levels were normalized to the housekeeping protein β**-**actin or GAPDH to adjust for variability of protein loading and expressed as a percentage of the vehicle control (as 100%). Cytosolic and nuclear protein was collected, following the manufacturer’s protocol in the Cytosolic and Nuclear protein extraction kit (Beyotime Institute of Biotechnology, Jiangsu, China). Briefly, to separate nuclear and cytosolic proteins, cells were washed and collected in PBS, centrifuged, resuspended in 200 μL of cytosolic protein extraction reagent A (containing 2 μL of PMSF), vortexed for 5 seconds, and incubated on ice for 15 min. Next, 10 μL of cytosolic protein extraction reagent B was added, and samples were vortexed for 5 seconds, incubated on ice for 1 min, and vortexed for 5 seconds. The supernatant, containing cytosolic proteins, was collected by centrifugation. The pellet was resuspended in nuclear extraction reagent (containing 2 μL of PMSF), vortexed for 30 seconds, incubated on ice for 30 min, and vortexed for 5 seconds every 1 to 2 min. The supernatant, containing nuclear proteins, was collected by centrifugation.

### Statistical analysis

All statistical analyses were performed using one-way ANOVA with repeated measures followed by Scheffe’s post hoc tests. Data were presented as mean ± standard error of the mean. A P value less than 0.05 was considered statistically significant.
